# Dynamic transcription profiles of “Qinguan” apple (*Malus* × *domestica*) leaves in response to *Marssonina coronaria* inoculation

**DOI:** 10.3389/fpls.2015.00842

**Published:** 2015-10-13

**Authors:** Jianhua Xu, Miaomiao Li, Peng Jiao, Hongxia Tao, Ningning Wei, Fengwang Ma, Junke Zhang

**Affiliations:** The Department of Pomology, College of Horticulture, Northwest A&F UniversityYangling, China

**Keywords:** apple, *M. coronaria*, transcriptome, disease resistance gene, cell components, polyphenol compounds

## Abstract

*Marssonina* apple blotch, caused by the fungus *Marssonina coronaria*, is one of the most destructive apple diseases in China and East Asia. A better understanding of the plant's response to fungi during pathogenesis is urgently needed to improve plant resistance and to breed resistant cultivars. To address this, the transcriptomes of “Qinguan” (a cultivar with high resistance to *M. coronaria*) apple leaves were sequenced at 12, 24, 48, and 72 h post-inoculation (hpi) with *Marssonina coronaria*. The comparative results showed that a total of 1956 genes were differentially expressed between the inoculated and control samples at the 4 time points. Gene ontology (GO) term enrichment analysis of differentially expressed genes (DEGs) revealed changes in cellular component, secondary metabolism including chalcone isomerase activity, phytoalexin biosynthetic process, anthocyanin-containing compound biosynthetic process, lignin biosynthetic process, positive regulation of flavonoid biosynthetic process; and molecular functions or biological processes related to the defense response, biotic stimulus response, wounding response and fungus response. Kyoto Encyclopedia of Genes and Genomes (KEGG) pathway analysis showed that DEGs were significantly enriched in flavonoid biosynthesis, vitamin B6 metabolism, phenylpropanoid biosynthesis, and the stilbenoid, diarylheptanoid and gingerol biosynthesis pathways. Furthermore, the importance of changes in cellular components and partial polyphenol compounds when encountering *M. coronaria* are discussed.

## Introduction

The domesticated apple (*Malus* × *domestica* Borkh.) is the main fruit crop in temperate regions of the world (Luby, 2003; Velasco et al., 2010). The *Marssonina* apple blotch caused by the fungus *Marssonina coronaria* is one of the most prevalent apple diseases in East Asia and China. Most of the widely-grown apple cultivars in China, such as “Fuji” and “Gala” apples, are highly susceptible to *M. coronaria*, while “Qinguan,” a local bred apple cultivar, has demonstrated high resistance with lower incidence of infection (Yin et al., 2013). However, the anti-fungal molecular mechanism in this cultivar remains unknown.

The exploration and application of resistance mechanisms in apple plants is very important for apple breeding. The conditions for *in vitro* culture and the conidia induction of *M. coronaria* have been developed and optimized (Lee et al., 2011). The method for *M. coronaria* resistance identification of different apple germplasm have been settled up by conidia suspension inoculation on detached leaves (Wang et al., 2012; Huang et al., 2013). The infection procedure and critical time points in *M. coronaria* pathogenesis have been clearly outlined by monitoring the symptoms on *in vitro* apple leaves with fluorescence and electron microscopy. Normally, the conidia germinated on the leaf surface of apple after 6 hpi, typical hypha penetration and haustoria formation was seen in epidermal cells within 24 hpi, and callose deposition was observed on the epidermal cell wall around the infection sites after 12 hpi. Colony can be found in the leaf tissue 3 days post inoculation, the subcutilar hyphal strands can be found expanding radically around the infection sites 5 days post inoculation and the acervuli can be seen on the leave tissue after 11 days post inoculation. The symptom of the disease on the leave surface including a round, brown lesion with yellow edge can be completely appeared about 15 days after inoculation. The growth and development of the pathogen were much slower in the resistant apple genotype than that of susceptible one while the callose deposition was the opposite (Wang et al., 2012; Zhao et al., 2013).

During pathogen invasion, plant responses varied from cell structure enhancement to synthesis of specialized chemical components. A common response by plants to fungal attack is deposition of callose, a (1,3)-β-glucan polymer, which provide a structural barrier to slow fungal penetration. Evidence showed that the transgenic *PMR4* Arabidopsis lines over expressing a callose synthase gene completely inhibit the penetration of powdery mildew (Dorothea et al., 2013). Although, the plant cell wall and its component associated with plant disease resistance have been discussed in high plants (Hückelhoven, 2007), but the resistance linked to apple cell wall have not been reported.

During pathogen invasion, antimicrobial substances of low molecular weight produced by plants were called Phytoalexins, which belongs to a large and diverse class of chemical compounds, including phenylpropanoid compounds, stilbenes, and flavonoids (Ferguson, 2001; Quideau et al., 2011). Polyphenols are important agents in defense against biotic or abiotic stressors, such as nutrient deficiency, drought, temperature variations, salt injury, and pathogen infection stress. These molecules also serve to facilitate communication between plants, or to transducte signals from the environment (Juszczuk et al., 2004; Bruno and Sparapano, 2006; Deytieux-Belleau et al., 2009; Saviranta et al., 2010). Study on phenylpropanoid compounds, stilbenes in apple are very few. And a large proportion of the published research on flavonoids in apples were focused on composition and content in peels and pulp. The importance of phenylpropanoids, stilbenes, and flavonoids in plant-pathogen interactions also has not been thoroughly studied.

Although, the exploration of resistance genes in apple have been started by identifying the genes responding to *M. coronaria* infection, the expression of some expressed sequence tags (ESTs) have been proved related to the pathogensis on apple leaves (Zhou et al., 2012), some proteins accumulated during *M. coronaria* infection were also identified using two-dimensional electrophoresis (2-DE) (Li et al., 2014), the exact resistance genes and the dynamic transcriptome changes and possible mechanisms behind this resistance have not been reported. This new dataset will provide useful information for future genetic and genomic studies of apple fungi disease resistance.

The purpose of this paper is to comprehensively compare the dynamic gene expression difference between the *M. coronaria* inoculated and control apple leaves after inoculation outline the genes participate in the fungal response of “Qinguan” apple during *M. coronaria* pathogensis, laying a firm foundation for elucidating the molecular basis behind the disease resistance.

## Materials and methods

### Plant material and *M. coronaria* inoculation

Four year old “Qinguan” apple trees were grown in a round pot (35 cm in radius and 45 cm in height) under a rain shelter at the experiment station of Northwest A. & F. University, Shaanxi, China. About 120~150 pieces of healthy mature “Qinguan” apple leaves of approximately the same size were detached on August 2nd when natural infection would happen. The collected leaves were immediately surface-sterilized with 8% sodium hypochlorite and then washed with autoclaved sterile water. After the leaf blades were air-dried, the ends of the petiole were wrapped with sterile water wetted absorbent cotton. Half of the sampled leaves were inoculated with *M. Coronaria* according to Wang et al. (2012). The other half were treated with distilled water as a control. All treated and control leaves were put into plastic trays and covered with transparent films. The leaves were cultured in an incubator at 25°C with a relative humidity of 95–100% and a natural photoperiod.

Pathogenesis on the leaf surface was monitored by microscope every 24 h to ensure successful inoculation and development of disease.

Thirty to Thirty five pieces of inoculated and control sample leaves were randomly harvested at 12, 24, 48, and 72 h post-inoculation (hpi) and marked as T1, T2, T3, T4, and CK1, CK2, CK3, CK4 accordingly. All treated samples were frozen immediately in liquid nitrogen and stored at −80°C until further processing.

### RNA isolation, library construction and RNA-sequencing

Total RNA from each sample was isolated separately from appropriate amount of mixed leave powder using the RN38 EASY spin plus Plant RNA kit (Aidlab Biotech, Beijing, China). Sequencing libraries were prepared using a New England Biolabs (NEB) Next® Ultra™ Directional RNA Library Prep Kit for Illumina® (Ipswich, MA, US) sequencing following the manufacturer's protocol. Sequencing was performed on an Illumina HiSeq™ 2000 sequencing platform in accordance with the manufacturer's instructions. An additional biological replicate of T2, T2 repeat (T2-r), was used to control for error.

### Sequence filter, assembly, mapping and gene annotation

Raw reads were filtered to obtain high-quality clean reads by trimming adaptor sequences, sequences mapping to more than one location in the genome, ambiguous reads, and low-quality reads (the reads in which the proportion of Ns is greater than 5%, with “N” representing unknown base identities). The clean reads were assembled to generate a non-redundant set of transcripts using the Trinity method (http://trinityrnaseq.sourceforge.net/) (Grabherr et al., 2011). The transcripts were clustered based on their nucleotide sequence identities. The longest transcripts in the cluster units were regarded as unigenes. The unigenes were mapped to the apple genome using Bowtie 2 software with default settings (Langmead and Salzberg, 2012).

To predict the function of the RNA sequence, the best hit against the *Malus* × *domestica* genome predicted genes was determined using six combined databases, including Swiss-prot, Arabidopsis homolog, TrEMBL homolog, Poplar homolog, Peach homolog, and Grape homolog. Sequences were blasted using BLASTP (TBLASTN for grape) with an *E*-value of 1.00E-6. When a gene had a match to Swiss-prot, it was assigned as the best match for that gene, followed in order by Arabidopsis homolog, TrEMBL homolog, Poplar homolog, Peach homolog and Grape homolog.

### Identification of DEGs, GO and pathway functional enrichment analysis of the DEGs

The expression levels of genes were normalized by FPKM (Mortazavi et al., 2008). EBSeq software was applied to perform a Chi-square test and then *P*-values were checked for false discovery rate (FDR) (Leng et al., 2013). Genes with FDR < 0.01 and fold change of FPKM ratio = 2 between inoculated and control were chosen as DEGs. A Venn diagram analysis of the total, downregulated and upregulated DEGs at the four time points was created online (http://bioinfogp.cnb.csic.es/tools/venny/index.html).

GO pathway enrichment analysis was utilized to identify significantly enriched functional classification or metabolic pathways in DEGs by Blast2GO with *E* < 1e-5 (Ashburner et al., 2000; Conesa et al., 2005). The significantly enriched pathways were identified with corrected *p* < 0.05.

The Kyoto Encyclopedia of Genes and Genomes (KEGG, http://www.genome.jp/kegg/) pathway tool is an alternative approach to understanding gene functions, with an emphasis on biochemical pathways. The assembled transcript sequences were searched against the KEGG database using BLASTX with a cut-off *E*-value of 1e-5. The significantly enriched pathways were identified as those with a *p* < 0.05 and a corrected *p* < 0.05.

### Data validation

To verify the reliability of the transcriptomic profiling data, the correlation between transcriptome sequencing data from sample T2 and its biological replicate T2-r was examined. The correlation of DEGs between T2 vs. CK2 and T2-r vs. CK2 was calculated by SPSS ver. 19.0 (IBM SPSS software) using the Pearson method with double-tail test.

## Results

### Deep sequencing and assembly

In total, 25.74 Gigabases were obtained by sequencing from 9 samples, averaging 2.86 Gigabases each, resulting in 254.85 million clean reads in total, and averaging 28.31 million clean reads for each sample. All RNA sequencing data and mapping results are presented in Table 1.

**Table 1:** **Mapping results of sequenced reads from inoculated and control transcriptomes**.

**Sample ID**	**Total clean reads**	**Total bases**	**Mapped reads numbers**	**Mapped reads percentage (%)**	**Unique mapped reads numbers**	**Unique mapped reads percentage (%)**
T1	26311896	2657276450	14108574	53.62	13449501	95.33
CK1	30880114	3118641764	16403578	53.12	15602836	95.12
T2	27680162	2795475956	14848885	53.64	14162508	95.38
T2-r	30935366	3123392151	16581431	53.60	15820223	95.41
CK2	24697402	2494256507	13718751	55.55	13093807	95.44
T3	28237484	2851667103	15046419	55.29	14332010	95.25
CK3	28811592	2909734246	15428864	53.55	14648881	94.94
T4	29594490	2988712362	15728014	53.15	14949219	95.05
CK4	27699322	2797379123	14632657	52.83	13871314	94.80
Total	254847828	25736535662	136497173	53.56	129930299	95.19

The correlation co-efficiency of FPKM between the two independent biological replicates T2 and T2-r was 0.738^**^, and the *p*-value was 0.000 (far less than 0.001). These results indicated that the two biological repeats were highly correlated.

### DEG identification

**Figure 1:**
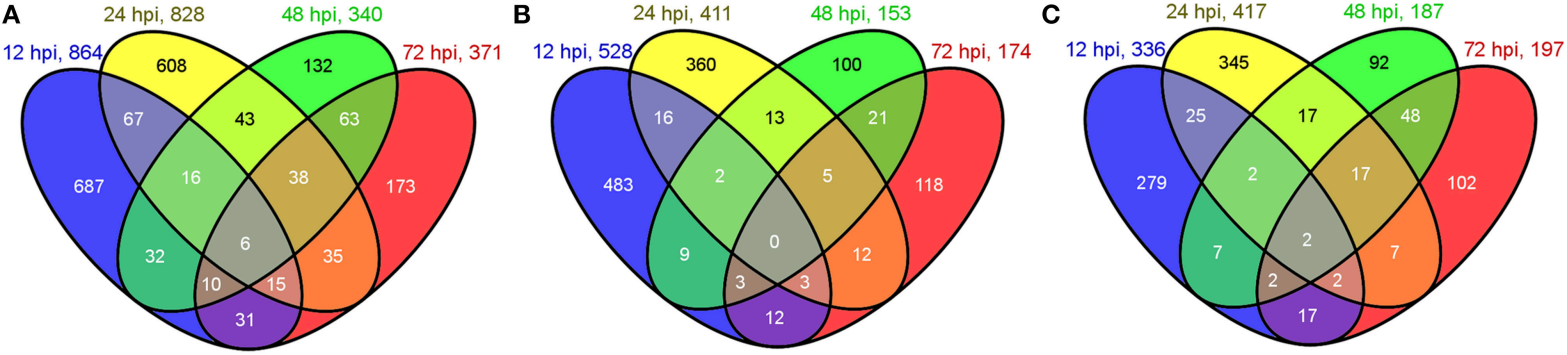
**Venn diagram of DEGs at 12, 24, 48, and 72 hpi**. **(A)** Numbers of total DEGs. **(B)** Numbers of upregulated DEGs. **(C)** Numbers of downregulated DEGs. Numbers in overlapping areas shows the shared DEGs at different time points.

In total, 1956 genes were identified as DEGs between the inoculations and their controls, including 864, 828, 340, and 371 genes at 12, 24, 48, and 72 hpi, respectively. A Venn diagram analysis of total DEGs at the four different time points is shown in Figure 1A. Among the total DEGs, 528, 411, 153, and 174 were upregulated (Figure 1B), whereas 336, 417, 187, and 197 were downregulated (Figure 1C) at 12, 24, 48, and 72 hpi, respectively. Among the 1956 DEGs, most of them showed 2~5 fold changes of their FPKM ratio (log2FC) between the inoculations and their controls, while only a small portion of DEGs were greatly induced (more than 5 folds) by the inoculation compared to their control. The greatly induced DEGs at different time points were list out in Supplementary Table S1.

### Dynamic changes of DEGs

The highest number of DEGs between the inoculated and control samples was detected at 12 hpi. The number of DEGs at 24 hpi was similar to that of 12 hpi, however, the number of DEGs at 48 and 72 hpi decreased to about 40% of those at 12 and 24 hpi. The rapid decrease of DEG number after 24 hpi indicates that the difference of induced gene differential expression compare to their control was narrowed down at 48 and 72 hpi.

**Figure 2:**
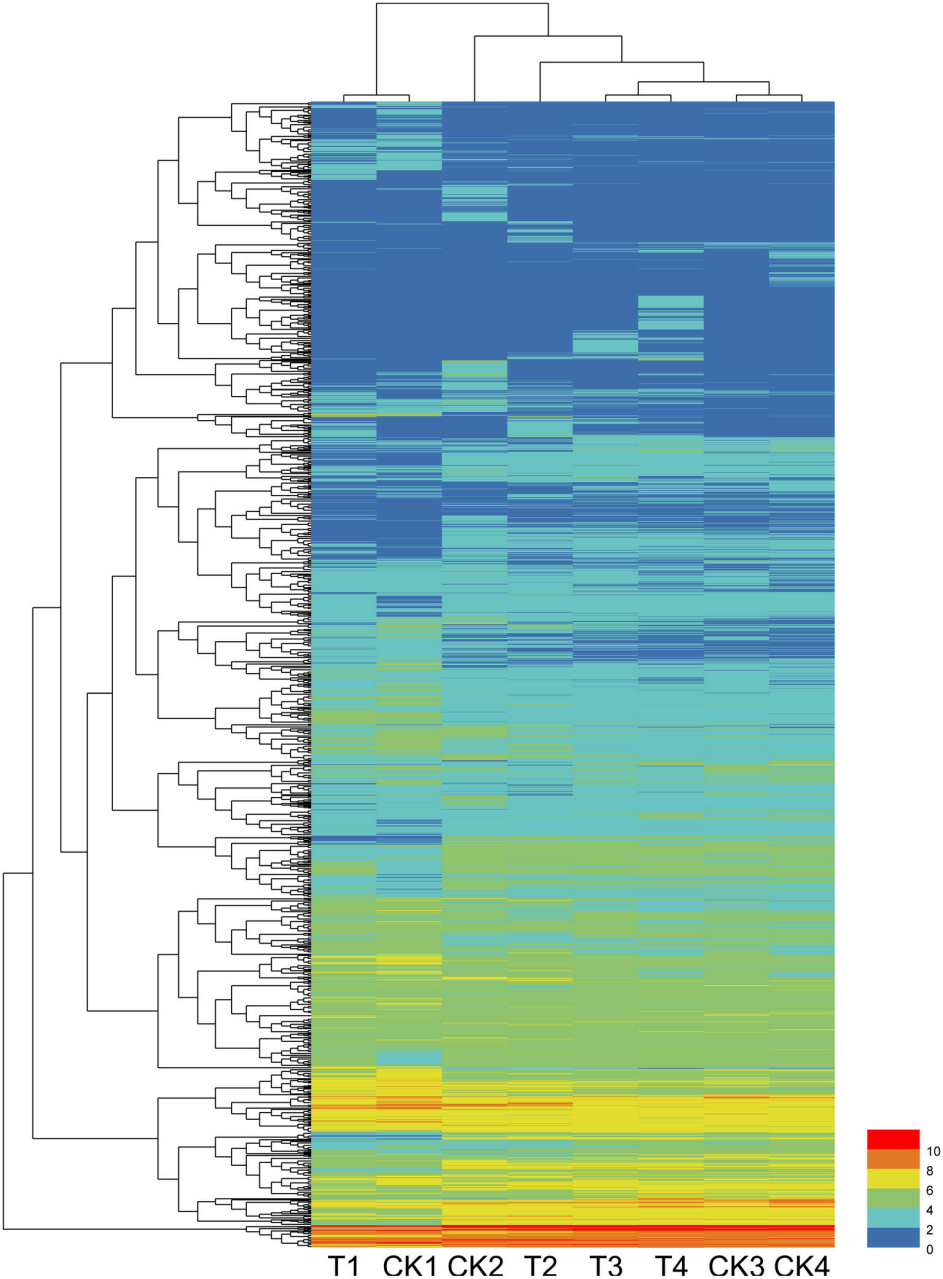
**Heatmap of DEGs expression of inoculation and control samples at 12, 24, 48, and 72 hpi**.

Among the total DEGs, 687, 608, 132, and 173 DEGs were only differentially expressed at one time points of 12, 24, 48, and 72 hpi, respectively, indicating that these genes only responded to the pathogen induction at a specific stage after inoculation. In contrast, 6 DEGs were found to be differentially expressed (up or down regulated compare to their control) at all 4 tested time points. These genes are as follows: Cysteine-rich repeat secretory protein 55 (MDP0000555329), and peroxidase (MDP0000770103), which were downregulated at all four time points; 60S ribosomal protein L5 (MDP0000150109), downregulated except for at 72 hpi; UDP-glucose 6-dehydrogenase (MDP0000193220), downregulated except for at 12 hpi; Scarecrow-like protein 4 (MDP0000575908), upregulated except for at 48 hpi; and chloroplast linoleate 13S-lipoxygenase 2-1 (LOX2) (MDP0000211556), upregulated except for at 12 hpi. Other DEGs were differentially expressed at two or three time points during pathogenesis and their number ranged from 10 to 67, accounting for less than 8% of the total DEGs. The dynamic expression change of the DEGs with the time point were shown in the hierarchical heatmap (Figure 2). This result implied that most of the DEGs are differentially expressed at one time point and their relay race may contribute to final resistance of this cultivar.

### GO assignments of DEGs

**Figure 3:**
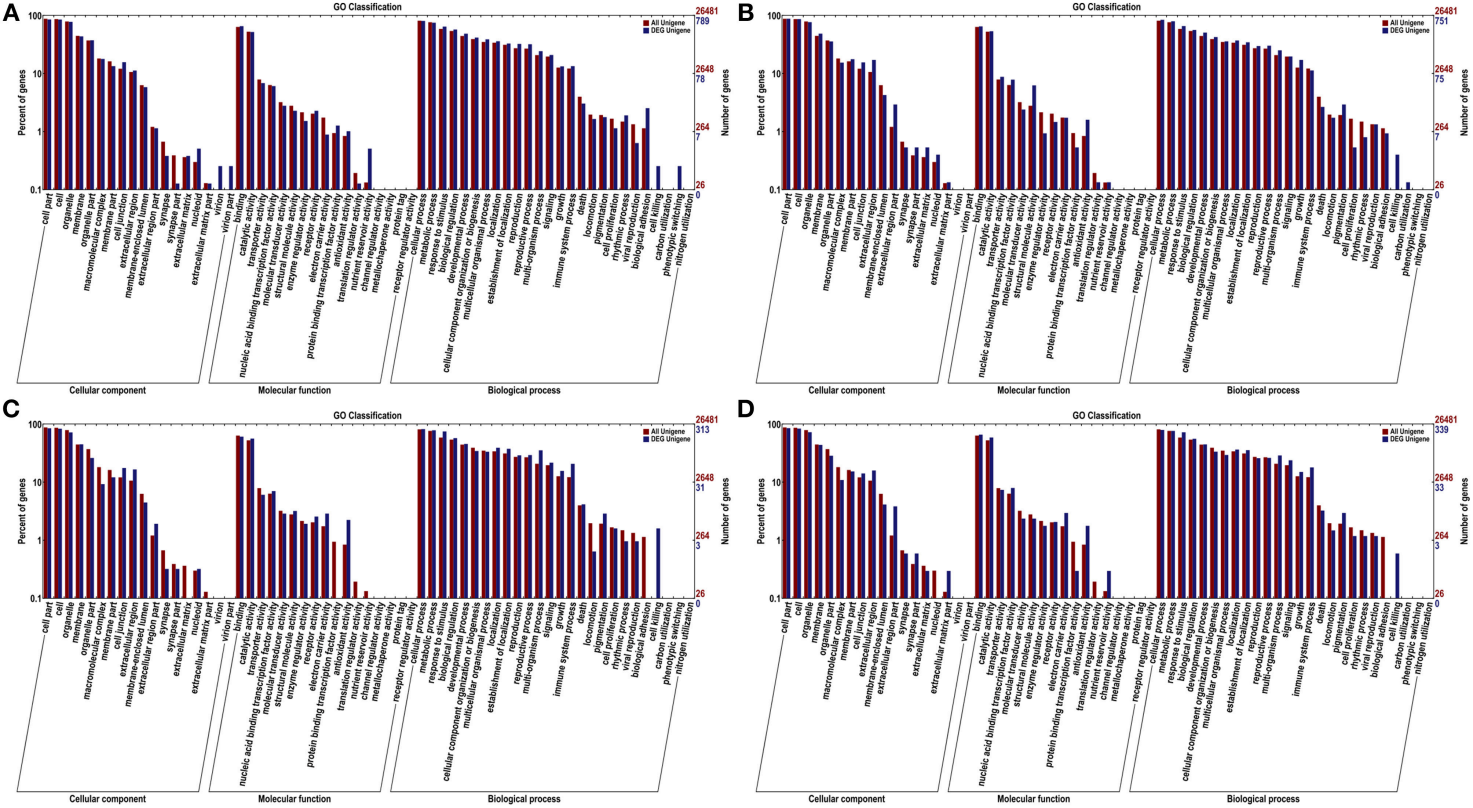
**GO categories of unigenes and DEGs at 12 hpi (A), 24 hpi (B), 48 hpi (C), and 72 hpi (D)**. The genes were functionally categorized into three groups; “cellular component,” “molecular function” and “biological process.” Here, two levels of the assignment results were plotted. Gene number percentages are on ordinate left, the number of genes are on the right.

To get an overview of the function category of the genes participated in the infection response, the DEGs between the inoculated and the control were classified by Gene Orthology. As a result, 789, 751, 313, and 339 DEGs were assigned at least one GO term at 12, 24, 48, and 72 hpi, respectively, including 61 functional groups at the second level (Figure 3). This result implies that a wide ranges of functional genes responsed to the pathogen inoculation.

At the first GO level, “cell,” “cell part,” “organelle,” “membrane” and “organelle part” terms were among the top five ranks in the cellular component category. For molecular function, “binding” and “catalytic activity” were the most abundant subcategories. While “cellular process,” “metabolic process” and “response to stimulus” were the most highly represented in the biological process category.

Aimed to find the most concentrated gene function groups in DEGs, the The significantly enriched GO terms of DEGs annotation at the four time points with corrected *p* < 0.05 are listed in Table 2 and the DEGs in significantly enriched GO terms are provided in Supplementary Table S2. Significantly enriched GO terms involved in “cellular component” were concentrated at the 12 and 24 hpi time points. No “cellular component” GO terms were significantly enriched at 48 or 72 hpi. This result implied that DEGs in “cellular component” category were greatly induced upon the pathogen inoculation.

**Table 2:** **GO terms significantly enriched in DEGs at four time points**.

**Time points**	**First level category**	**GO term**	**GO ID**	**Cluster frequency (%)**	**Corrected *p*-value**
12 hpi	Cellular component	Cytosol	GO:0005829	183 out of 707 (25.88)	6.30e-03
		COPII vesicle coat	GO:0030127	5 out of 707 (0.71)	1.21e-02
		1,3-beta-D-glucan synthase complex	GO:0000148	6 out of 707 (0.85)	1.27e-02
		Plasmodesma	GO:0009506	123 out of 707 (17.4)	2.55e-02
	Molecular function	Cadmium ion transmembrane transporter activity	GO:0015086	10 out of 644 (1.55)	1.64e-04
		Organic phosphonate transmembrane-transporting ATPase activity	GO:0015416	10 out of 644 (1.55)	4.08e-04
		Lupeol synthase activity	GO:0042299	9 out of 644 (1.40)	1.68e-03
		ARF guanyl-nucleotide exchange factor activity	GO:0005086	5 out of 644 (0.78)	2.02e-02
		1,3-beta-D-glucan synthase activity	GO:0003843	6 out of 644 (0.93)	3.14e-02
		ATPase activity, coupled to transmembrane movement of substances	GO:0042626	13 out of 644 (2.02)	3.98e-02
	Biological process	Vegetative to reproductive phase transition of meristem	GO:0010228	53 out of 727 (7.29)	1.27e-05
		Lead ion transport	GO:0015692	11 out of 727 (1.51)	2.29e-05
		Cellular response to indolebutyric acid stimulus	GO:0071366	10 out of 727 (1.38)	9.18e-05
		Abscisic acid transport	GO:0080168	11 out of 727 (1.51)	1.05e-03
		Protein targeting to vacuole involved in ubiquitin-dependent protein catabolic process via the multivesicular body sorting pathway	GO:0043328	4 out of 727 (0.55)	1.37e-03
		Defense response by callose deposition in cell wall	GO:0052544	18 out of 727 (2.48)	5.03e-03
		Regulation of cell differentiation	GO:0045595	17 out of 727 (2.34)	5.64e-03
		Indole glucosinolate catabolic process	GO:0042344	10 out of 727 (1.38)	1.39e-02
		Defense response	GO:0006952	62 out of 727 (8.53)	1.76e-02
		Trichome morphogenesis	GO:0010090	29 out of 727 (3.99)	3.34e-02
		Drug transmembrane transport	GO:0006855	13 out of 727 (1.79)	3.48e-02
24 hpi	Cellular component	Cell wall	GO:0005618	68 out of 693 (3.81)	2.68e-06
		Extracellular region	GO:0005576	89 out of 693 (12.84)	3.22e-06
		Plant-type cell wall	GO:0009505	59 out of 693 (8.51)	4.55e-06
		Extracellular region part	GO:0044421	14 out of 693 (2.02)	1.07e-04
		Anchored component of plasma membrane	GO:0046658	22 out of 693 (3.17)	4.94e-04
		Anchored component of membrane	GO:0031225	15 out of 693 (2.16)	7.63e-03
		Polyketide synthase complex	GO:0034081	4 out of 693 (0.58)	1.05e-02
	Molecular function	Structural constituent of cell wall	GO:0005199	11 out of 644 (1.71)	2.41e-05
		Chalcone isomerase activity	GO:0045430	5 out of 644 (0.78)	5.06e-03
		2,3,4,5-tetrahydropyridine-2,6-dicarboxylate N-succinyltransferase activity	GO:0008666	4 out of 644 (0.62)	1.42e-02
		Biphenyl synthase activity	GO:0033815	4 out of 644 (0.62)	1.42e-02
	Biological process	Syncytium formation	GO:0006949	14 out of 705 (1.99)	8.78e-07
		Plant-type cell wall loosening	GO:0009828	12 out of 705 (1.70)	1.50e-06
		Plant-type cell wall modification involved in multidimensional cell growth	GO:0009831	11 out of 705 (1.56)	3.05e-06
		Regulation of cell size	GO:0008361	28 out of 705 (3.97)	9.33e-04
		Polyamine catabolic process	GO:0006598	21 out of 705 (2.98)	3.32e-03
		Response to karrikin	GO:0080167	36 out of 705 (5.11)	5.22e-03
		Auxin polar transport	GO:0009926	23 out of 705 (3.26)	2.01e-02
		Pattern specification process	GO:0007389	29 out of 705 (4.11)	2.44e-02
		Cellular modified amino acid biosynthetic process	GO:0042398	19 out of 705 (2.70)	2.82e-02
		Response to wounding	GO:0009611	63 out of 705 (8.94)	3.12e-02
		2-hydroxybenzoyl-CoA catabolic process	GO:1901886	4 out of 705 (0.57)	3.72e-02
		4-hydroxycoumarin biosynthetic process	GO:1901884	4 out of 705 (0.57)	3.72e-02
		Phytoalexin biosynthetic process	GO:0052315	4 out of 705 (0.57)	3.72e-02
48 hpi	Biological process	Response to UV-B	GO:0010224	26 out of 298 (8.72)	1.66e-07
		Response to wounding	GO:0009611	44 out of 298 (14.77)	3.96e-07
		Response to biotic stimulus	GO:0009607	11 out of 298 (3.69)	3.33e-06
		Positive regulation of flavonoid biosynthetic process	GO:0009963	19 out of 298 (6.38)	3.47e-05
		Defense response to fungus, incompatible interaction	GO:0009817	42 out of 298 (14.09)	4.03e-05
		Regulation of plant-type hypersensitive response	GO:0010363	39 out of 298 (13.09)	4.20e-05
		Coumarin biosynthetic process	GO:0009805	18 out of 298 (6.04)	5.51e-05
		Response to karrikin	GO:0080167	23 out of 298 (7.72)	2.51e-04
		Killing of cells of other organism	GO:0031640	5 out of 298 (1.68)	3.42e-04
		Defense response	GO:0006952	36 out of 298 (12.08)	4.28e-04
		Cellular modified amino acid biosynthetic process	GO:0042398	14 out of 298 (4.70)	4.88e-04
		Polyamine catabolic process	GO:0006598	14 out of 298 (4.70)	6.82e-04
		Protein targeting to membrane	GO:0006612	35 out of 298 (11.74)	9.09e-04
		Anthocyanin-containing compound biosynthetic process	GO:0009718	12 out of 298 (4.03)	2.48e-03
		Response to chitin	GO:0010200	35 out of 298 (11.74)	6.39e-03
		Lignin biosynthetic process	GO:0009809	13 out of 298 (4.36)	1.25e-02
72 hpi	Molecular function	Hydrolase activity, hydrolyzing O-glycosyl compounds	GO:0004553	11 out of 289 (3.81)	1.57e-02
		Metal ion binding	GO:0046872	37 out of 289 (12.80)	7.42e-02
	Biological process	Response to biotic stimulus	GO:0009607	11 out of 317 (3.47)	6.43e-06
		Regulation of plant-type hypersensitive response	GO:0010363	40 out of 317 (12.62)	7.87e-05
		Protein targeting to membrane	GO:0006612	38 out of 317 (11.99)	1.88e-04
		Positive regulation of flavonoid biosynthetic process	GO:0009963	16 out of 317 (5.95)	8.95e-03
		Response to chitin	GO:0010200	36 out of 317 (11.36)	1.02e-02
		Defense response to fungus	GO:0050832	37 out of 317 (11.67)	2.29e-02
		Systemic acquired resistance, salicylic acid mediated signaling pathway	GO:0009862	24 out of 317 (7.57)	4.00e-02

Significantly, enriched GO terms involved in “molecular function” and “biological process” were those related to defense, biotic stimulus, wounding and fungus responses (GO:0006952, GO:0009607, GO:0009611, GO:0050832, GO:0009817). In addition, a variety of genes related to secondary products accumulation were significantly enriched, including chalcone isomerase activity (GO:0045430), phytoalexin biosynthetic process (GO:0052315), anthocyanin-containing compound biosynthetic process (GO:0009718), lignin biosynthetic process (GO:0009809), and positive regulation of flavonoid biosynthetic process (GO:0009963). Although the GO terms of DEGs in the “molecular function” and “biological process” category were significantly enriched in first level, it was difficult to address the fluction of the DEGs in subcategory.

### Significantly enriched KEGG pathways in DEGs

To find the most differentially induced pathways, KEGG pathways were identified according to DEGs with a corrected *p* < 0.05 at various time points. Four secondary metabolic pathways, (1) flavonoid biosynthesis, (2) vitamin B6 metabolism, (3) phenylpropanoid biosynthesis, and (4) the stilbenoid, diarylheptanoid, and gingerol biosynthesis were significantly enriched at 24, 48 or 72 hpi (corrected *p*-values of all KEGG pathways at 12 hpi were > 0.05) (Table 3) and the DEGs which were significantly enriched in KEGG pathways are listed in Supplementary Table S3. No pathway was significantly enriched at 12 hpi. This result implied that secondary metabolic pathway especially flavonoid biosynthesis were the most greatly induced pathway at 12, 48 and 72 hpi and stilbenoid, diarylheptanoid, and gingerol biosynthesis pathway were the most greatly induced pathway at 48 and 72 hpi.

**Table 3:** **Significantly enriched KEGG pathways of DEGs at 12, 24, 48, and 72 hpi**.

**Time Point**	**Pathway ID**	**Pathway**	**DEG number with pathway annotation**	**Percentage of DEGs with pathway annotation**	***P*-value**	**Corrected *p*-value**
24	ko00941	Flavonoid biosynthesis	15	9.26	2.31E-10	1.68e-08
	ko00750	Vitamin B6 metabolism	5	3.09	3.04E-04	2.22e-02
48	ko00940	Phenylpropanoid biosynthesis	14	23.73	5.16E-11	2.84e-09
	ko00941	Flavonoid biosynthesis	9	15.25	1.71E-08	9.38e-07
	ko00945	Stilbenoid, diarylheptanoid and gingerol biosynthesis	4	6.78	3.67E-04	2.02e-02
72	ko00940	Phenylpropanoid biosynthesis	12	19.67	1.35E-08	7.80e-07
	ko00941	Flavonoid biosynthesis	5	8.2	6.62E-04	3.84e-02
	ko00945	Stilbenoid, diarylheptanoid and gingerol biosynthesis	4	6.56	4.18E-04	2.42e-02

## Discussion

### Significantly enriched GO terms in cellular components and disease resistance

In this study, the DEGs of significantly enriched GO terms involved in genes related to cellular components contained coat protein complexes (COPII vesicle coat), plasmodesma, cell wall, and 1,3-beta-D-glucan synthase complex. This implied the genes related to subcellular structure change or cell wall enhancement were induced in resistant apple leaves of “Qinguan” when encountering *M. coronaria* infection.

Vesicles are responsible for inner cellular transport between organelles of the endomembrane system (endoplasmic reticulum, Golgi apparatus, endosomes, lysosomes and vacuoles), and between the plasma membrane (Robinson et al., 1998; Surpin and Raikhel, 2004). Vesicle trafficking is linked to the regulation of immune signaling (Stegmann et al., 2012). Mutations in *AtVAMP7C* gene, which is thought to direct fusion between the vacuolar membrane and vesicle, resulted in increased salt tolerance (Leshem et al., 2006). Vesicle formation and budding are a consequence of the continued assembly of the coat proteins. The vesicle coat protein complexes include the clathrin coat protein complex-I (COPI) and coat protein complex-II (COPII) (Bickford et al., 2004; Lee et al., 2004; Edeling et al., 2006). The COPII vesicle coat coordinates the budding of transport vesicles from the endoplasmic reticulum in the initial step of the secretory pathway (Bickford et al., 2004). ADP-ribosylation factor (ARF) families play important roles in vesicle-associated processes, ranging from vesicle formation and transport to exocytosis (Bos et al., 2007). In the present study, the differentially expressed genes involved in the cellular component COPII vesicle coat (GO: 0030127) were upregulated at 12 hpi. Coinciding with this change, genes involved in ADP-ribosylation factor (ARF) guanyl-nucleotide exchange factor activity (GO: 0005086) were also upregulated at 12 hpi in the category of molecular function. The significantly enriched GO terms, “COPII vesicle coat (GO: 0030127),” provided strong evidence that vesicle trafficking took part in plant immune system when encountering biotic stress.

Plasmodesma is a super cellular structure of plant (Lucas, 1995). It provides a direct cell-to-cell cytoplasmic pathway for material transport and message transference, and converts the colonies of independent cells into an interconnected symplast system, playing important roles in plant growth and development, as well as in the response and adaptation of plants to environmental changes (Carrington et al., 1996; Jacinto et al., 1997; Ding, 1998). Among the 123 genes involved in the cellular component plasmodesma (GO: 0009506), 89 genes were upregulated at 12 hpi. The changes of COPII and plasmodesma related DEGs implied that activities related to this cell component were enhanced and COPII and plasmodesma might be ready for material transport.

The resistance mediated by chemical composition changes or physical structure modification of plant cell walls can be an effective physical barrier to pathogens. Deposition of callose, accumulation of phenol compounds, and lignin synthesis increases the structural strength of cell walls following the invasion of pathogens (Farmer, 1985; Grand et al., 1987; Hückelhoven, 2007). Callose, a β-1, 3-D-glucan, exists widely in plant cell walls and plasma membrane. At sites of attempted penetration by fungal pathogens, glucan is a major structural component of papillae in epidermal cells (Enkerli et al., 1997). After Japanese pear leaves were inoculated with *Alternaria alternata*, glucan deposition was observed as papillae at the infection sites in abaxial epidermis (Suzuki et al., 2003). After inoculation with powdery mildew species, *Golovinomyces orontii*, callose accumulation was observed at attempted infection sites (Jacobs, 2003). In this database, 1,3-beta-D-glucan synthase complex (GO:0000148), 1,3-beta-D-glucan synthase activity (GO:0003843) and callose deposition in cell wall (GO:0052544) were highly upregulated at 12 hpi. In addition, genes involved in plant-type cell wall loosening (GO:0009828) were downregulated at 12 hpi. The distinguishable GO terms involved in glucan deposition were strongly indicating glucan deposition played an important role when “Qinguan” encountered *M. coronaria*.

### Significantly enriched KEGG pathways and disease resistance

Phenylpropanoid compounds are natural products derived from cinnamic acid which is formed from phenylalanine via deamination by phenylalanine ammonia-lyase (PAL). From the major biosynthetic routes to the various classes of phenylpropanoids, we can realize phenylpropanoid pathway is a “core” pathway for the formation of monolignols/lignin, coumarins, benzoic acids, stilbenes, and flavonoids/isoflavonoids (Dixon and Paiva, 1995; Dixon et al., 2002).

Stilbenes are a small family of plant secondary metabolites derived from the phenylpropanoid pathway. The most intensively studied biological property of plant-produced stilbenes is their antifungal activity (Hart, 1981; Morales et al., 2000). Stilbene synthase, also termed resveratrol synthase, is a key enzyme in biosynthesis of stilbene-type phytoalexins. The genes coding for stilbene synthases have been transferred into tobacco (Hain et al., 1990, 1993), oilseed rape (Thomzik, 1993), rice (Stark-Lorenzen et al., 1997), barley and wheat (Leckband and Lörz, 1998) and such transgenic plants showed a significant increase in disease resistance. Resveratrol exogenously applied to apples inhibited *Venturia inaequalis* (the causal agent of apple scab) penetration of cuticular membranes (Schulze et al., 2005). In addition, the researchers also found a positive correlation between stilbene content and disease resistance (Langcake et al., 1979; Fornara et al., 2008; Schnee et al., 2008). The mechanism by which stilbenes inhibit fungi is not well understood.

Flavonoids are a diverse group of secondary metabolites that can be divided into subgroups, including anthocyanidins, flavonols, flavones, flavanols, flavanones, chalcones, dihydrochalcones, and dihydroflavonols (Treutter, 2006). Flavonoids and related phenolics have indisputable functions in protecting plants from fungal infection. Defense-related flavonoids can be divided into two types: pre-existing and stimulating. Pre-existing flavonoids are usually stored in particular locations and can be used as signaling molecules (Treutter, 2006) or are involved in various interactions between the plant and a pathogen (Schlösser, 1994). Physical injury, infection, or stress can stimulate the formation of flavonoids called phytoalexins (Treutter, 2006). The role of flavonoids against pathogens may be related to the following mechanisms: antioxidant properties; cross-linking of microbial enzymes; inhibition of microbial cellulases, xylanases, and pectinases; chelation of metals necessary for enzyme activity; storage in specialized cells from which they can be infused into attacked tissue (such as xylem vessels); formation of a hard, almost crystalline structure as a physical barrier against pathogen attack; and promotion of the formation of calli and tyloses, thus closing vessels and blocking aggressive invaders (Skadhauge et al., 1997; Beckman, 2000).

In the present study, the KEGG pathways “Phenylpropanoid biosynthesis (ko00940)” and “Stilbenoid, diarylheptanoid, and gingerol biosynthesis (ko00945)” were significantly enriched at 48 and 72 hpi, and “flavenoid biosynthesis (ko00974)” was significantly enriched at 24, 48, and 72 hpi. In addition, the GO term “Positive regulation of flavonoid biosynthetic process (GO:0009963)” was significantly enriched at 48 hpi and 72 hpi. This provides researchers with new ideas to enhance disease resistance, and the DEGs in these pathways should have more focused attention in future research.

This work presents the first transcriptome sequencing analysis of the “Qinguan” apple leaf inoculated with *M. Coronaria* vs. control at four time points post-inoculation. In the present study, a total of 1956 genes were identified as DEGs, including 864, 828, 340, and 371 genes at 12, 24, 48, and 72 hpi, respectively. GO and KEGG analyses of DEGs suggested that the mechanism of defense against *M. Coronaria* is very complex and involves multiple processes. These analyses revealed that (i) changes in cellular components, development of a resistant structure; (ii) synthesis of many secondary products, including phytoalexin, anthocyanin, lignin, flavonoid, phenylpropanoid, and stilbenoid; (iii) and responses to defense, biotic stimulus, wounding and fungus were involved in the plant actions.

It is rather difficult to identify the disease resistance gene in perennial woody apple plant for their complex genetic background. Normally, disease resistance of different cultivar in woody plant always accompany by other traits encoded by non resistance genes. Transcriptome comparison during pathogensis between different cultivars always harvests a large number of DEGs but it is difficult to identify the resistance related gene from the rest. A ideal strategy is the use of disease resistance mutant, but this kind of material is real rare in apple plant. A reasonable way of resistance gene identification is to screen the DEGs between the pathogen inoculated and control in the resistant cultivar, then mine the pathogen responsed DEGs by GO and pathway, finally confirm the resistance gene by expression comparison in resistance and susceptible cultivar. This strategy was used in present research, DEGs responsed to pathogen inoculation were identified from the resistant cultivar “Qinguan” apple and were filtered by GO and pathway, some related pathway and DEGs related pathogen induction were found, but the exact resistance genes and their function still need further identification.

## Author contributions

JX and ML were responsible for the inoculation and sequence analysis. JX also wrote the manuscript. PJ prepared cDNA samples for sequencing. HT and NW helped with data interpretation. FM and JZ designed the experiment, provided guidance on the whole study and contributed with valuable discussions. All authors have read and approved the manuscript.

### Conflict of interest statement

The authors declare that the research was conducted in the absence of any commercial or financial relationships that could be construed as a potential conflict of interest.
